# Costly neighbours: Heterospecific competitive interactions increase metabolic rates in dominant species

**DOI:** 10.1038/s41598-017-05485-9

**Published:** 2017-07-12

**Authors:** Matouš Janča, Lumír Gvoždík

**Affiliations:** 1 Department of Botany and Zoology, Masaryk University, Kotlářská 267/2, 611 37 Brno, Czech Republic; 2 Institute of Vertebrate Biology, Czech Academy of Sciences, Květná 8, 60365 Brno, Czech Republic

## Abstract

The energy costs of self-maintenance (standard metabolic rate, SMR) vary substantially among individuals within a population. Despite the importance of SMR for understanding life history strategies, ecological sources of SMR variation remain only partially understood. Stress-mediated increases in SMR are common in subordinate individuals within a population, while the direction and magnitude of the SMR shift induced by interspecific competitive interactions is largely unknown. Using laboratory experiments, we examined the influence of con- and heterospecific pairing on SMR, spontaneous activity, and somatic growth rates in the sympatrically living juvenile newts *Ichthyosaura alpestris* and *Lissotriton vulgaris*. The experimental pairing had little influence on SMR and growth rates in the smaller species, *L. vulgaris*. Individuals exposed to con- and heterospecific interactions were more active than individually reared newts. In the larger species, *I. alpestris*, heterospecific interactions induced SMR to increase beyond values of individually reared counterparts. Individuals from heterospecific pairs and larger conspecifics grew faster than did newts in other groups. The plastic shift in SMR was independent of the variation in growth rate and activity level. These results reveal a new source of individual SMR variation and potential costs of co-occurrence in ecologically similar taxa.

## Introduction

The energy costs of living constitute an important component of life histories. These costs, consisting in the minimum energy requirements of a resting postabsorptive individual, are termed the basal metabolic rate in endotherms or standard metabolic rate (SMR) in ectotherms. According to the increased intake hypothesis^1, 2^, a higher SMR is typical for active individuals with relatively larger internal organs^3^. Such individuals should be favoured under conditions with good availability of resources^4^. If resources are limited, however, then more energy is spent on maintenance (i.e. up to 50% of total energy budget^5^), and that reduces the energy that can be invested into somatic growth, reproduction, and survival (the compensation hypothesis). Empirical results have supported both hypotheses^6, 7^. Despite its context-dependency, SMR is linked with the fitness of individuals within a population^8^.

SMR usually varies two- or threefold within a population even after accounting for body size and temperature^9, 10^. Both intrinsic and extrinsic factors contribute to this variation, and these range from genetic and maternal influences to shelter availability and to social interactions^8^. Social interactions affect SMR immediately (phenotypic flexibility)^11, 12^ or as a result of the prolonged competition for resources (phenotypic plasticity) within or between species^13, 14^. Behaviourally interacting individuals shift their SMR according to their body size differences, (i.e. the presence of a bigger individual increases SMR in smaller conspecifics)^12^, and that may result from a hormonal stress response^15^. At the population level, metabolic rates negatively correlate with population density, and thus competition strength, across taxa^14^. Likely mechanisms causing this include a competition-induced shift in foraging as well as activity rates. Accordingly, the density-dependence of metabolic rates may affect the energy budget of individuals and population dynamics.

Competition-induced plastic responses in SMR have been studied mainly in conspecifics. This is surprising, because individuals interact not only with members of their own species but also with other species within an ecosystem. The intensity and costs of heterospecific competitive interactions are often similar to those accompanying conspecific interactions^16, 17^. In addition, the exposure to con- and heterospecifics can induce similar hormonal responses^18^. However, the relative contribution of con- or heterospecific competitive interactions to SMR variation remains unknown.

Here, we examine the influence of con- and heterospecific pairing on SMR in juvenile newts of the species *Ichthyosaura alpestris* and *Lissotriton vulgaris*. Larvae of both species frequently develop in the same water body^19^, and so freshly metamorphosed juveniles may interact in terrestrial shelters located near water^20, 21^. It could be expected that prolonged exposure to con- and heterospecific competitive interactions would induce plastic responses in SMR. Due to greater size differences and relative competitive abilities between species as compared to within species, we predicted a more pronounced SMR shift in heterospecific than in conspecific pairs. Specifically, we predicted that con- and heterospecific pairing would increase SMR in smaller individuals. If the competition-induced shift in SMR is mediated by increased foraging rates and activity levels^14^, then SMR should be positively associated with spontaneous locomotor activity (the increased intake hypothesis). Finally, because juvenile SMR are often confounded by the metabolic costs of growth^22^, we also measured their growth rates. In the case of association between the two traits, this allows for the comparison of SMR relative to growth rate in each group.

## Results

**Figure 1:**
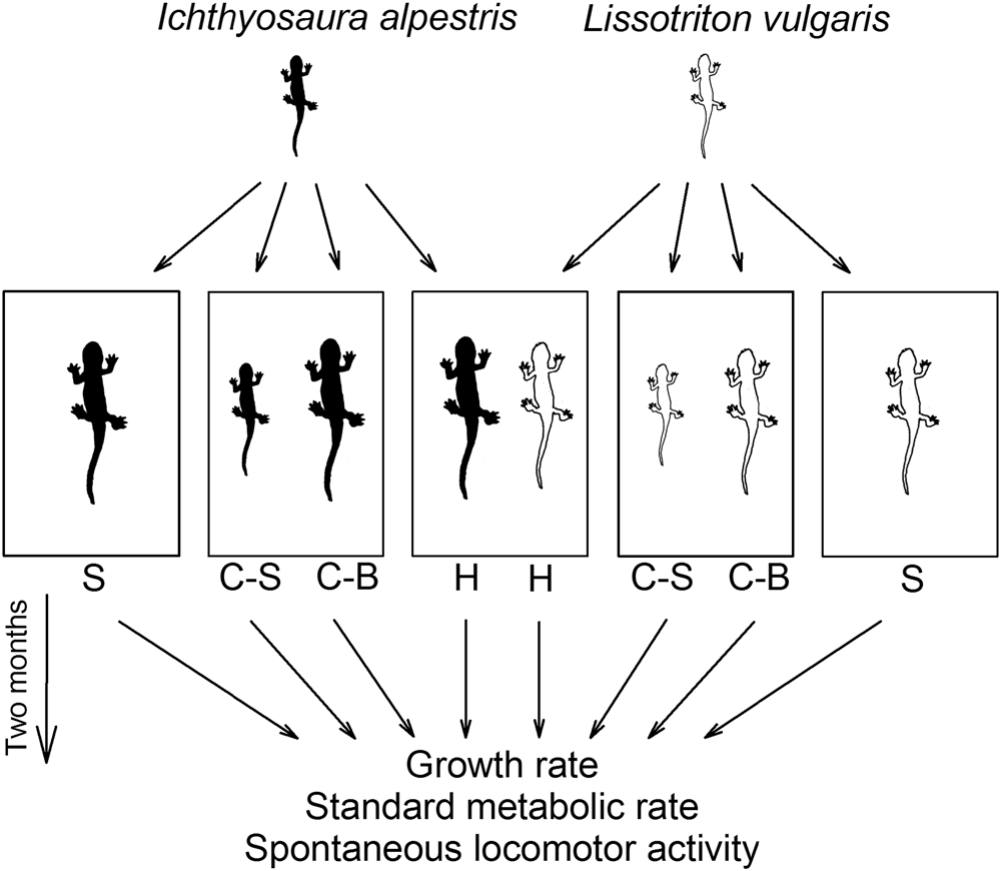
Schematic representation of the experimental design used. Juvenile newts were distributed among tanks separately or in con- and heterospecific pairs. In conspecific pairs, individuals were grouped according to their body size. Note that juveniles of *I. alpestris* were bigger than those of *L. vulgaris*. After two months, newt growth rates, standard metabolic rates, and spontaneous locomotor activity were measured at 18 °C. C-B, bigger conspecifics; C-S, smaller conspecifics; H, individuals from heterospecific pairs; S, singles.

**Figure 2:**
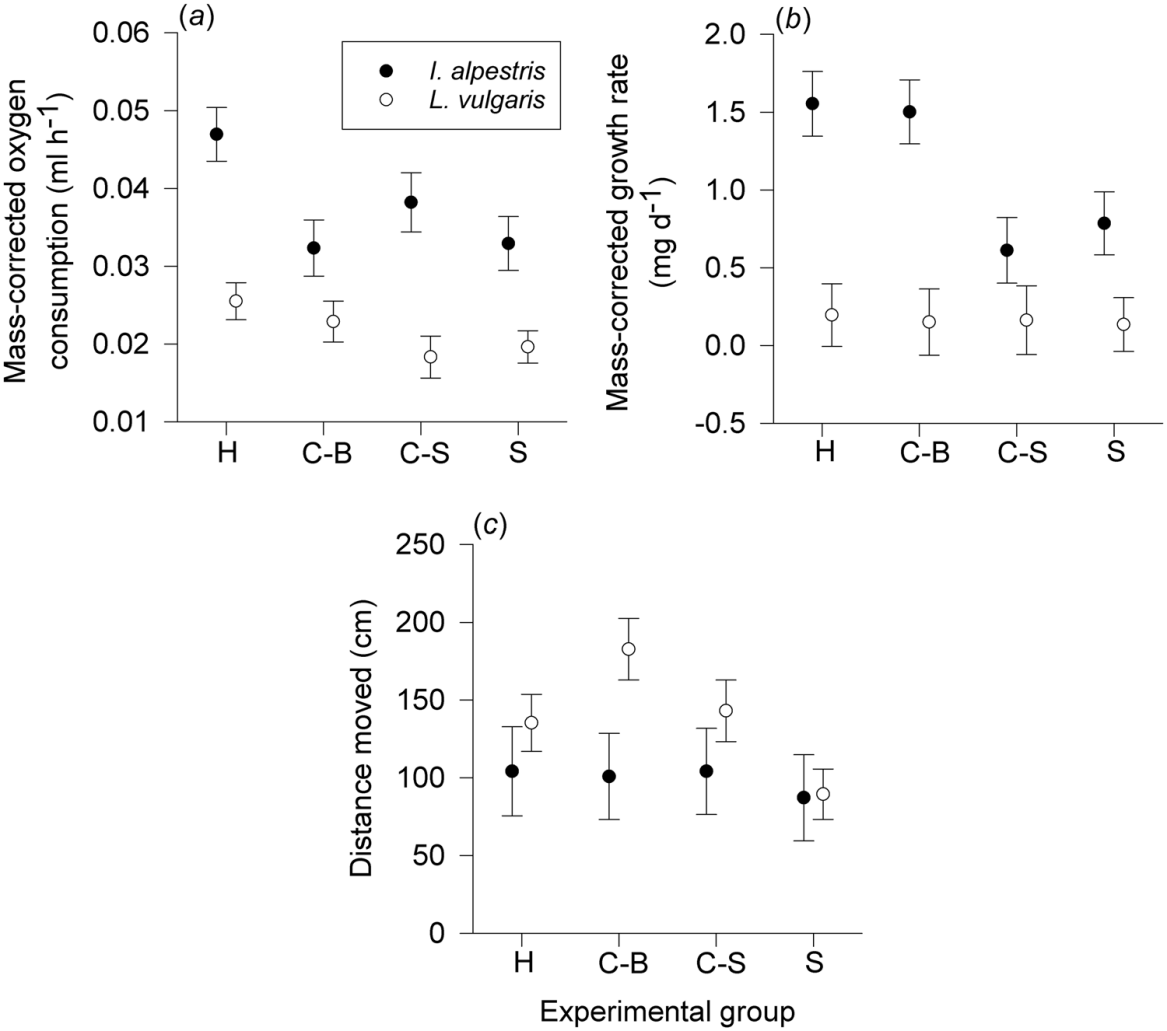
Influence of con- and heterospecific competitive interactions on newt traits. (**a**) Standard metabolic rates (minimum oxygen consumption), (**b**) somatic growth rates ([final body mass − initial body mass]/number of days of the experiment), and (**c**) spontaneous locomotor activity (distance moved during 30 min trial) in the juvenile newts *Ichthyosaura alpestris* and *Lissotriton vulgaris*. Metabolic and growth rates are body size-corrected means from a general linear model. Values are means ± s.e.m. C-B, bigger conspecifics; C-S, smaller conspecifics; H, individuals from heterospecific pairs; S, singles.

Standard metabolic rates were measured in juvenile newts of two different species experimentally exposed to the presence of con- and/or heterospecifics for two months (Fig. 1). Experimental pairing affected the SMR of *I. alpestris* (*F*_3,49_ = 4.19, *P* = 0.01; Supplementary Table S1). Specifically, *I. alpestris* from heterospecific pairs had higher maintenance costs than did larger conspecifics and newts reared individually (conspecifics: *t*_24_ = 2.40, *P* = 0.03; singles: *t*_24_ = 2.30, *P* = 0.03; Fig. 2a). In *L. vulgaris*, the influence of experimental pairing on SMR was statistically non-significant (*F*_3,47_ = 1.71, *P* = 0.18).

As with SMR, experimental pairing influenced growth rates in *I. alpestris* (*F*_3,54_ = 5.40, *P* = 0.002). Over two months’ time, individuals from heterospecific pairs and larger individuals from conspecific pairs grew faster than did newts reared individually and smaller conspecifics (heterospecific: *t*_26_ = 2.29, *P* = 0.03 and *t*_26_ = 2.69, *P* = 0.01; bigger conspecifics: *t*_26_ = 3.68, *P* = 0.0006 and *t*_26_ = 3.27, *P* = 0.002; Fig. 2b). In *L. vulgaris*, juveniles grew at similar rates in all groups (*F*_3,51_ = 0.02, *P* = 0.99). The association between growth rates and SMR was statistically non-significant in all groups (Supplementary Table S2).

Spontaneous locomotor activity measured at the end of competition trials was affected by experimental pairing only in *L. vulgaris* (*F*_3,52_ = 4.60, *P* = 0.007; *I. alpestris*: *F*_3,55_ = 0.69, *P* = 0.69). Specifically, individuals from conspecific pairs covered longer distances than did juveniles reared separately (larger conspecifics: *t*_28_ = 3.27, *P* = 0.003; smaller conspecifics: *t*_28_ = 2.41, *P* = 0.03; Fig. 2c). The association between locomotor activity and SMR was inconclusive in both species (Supplementary Table S2). Descriptive statistics for the measured traits are presented in Table S3.

## Discussion

Individual variation in SMR is affected by various intrinsic and extrinsic factors, including the social environment. Our study demonstrated that two-month exposure to competitive interactions affected SMR in heterospecific but not conspecific pairs in newts. Contrary to our prediction, the plastic shift occurred only in the larger (dominant) species. The SMR shift was largely independent of competition-induced variation in growth rates and spontaneous locomotor activity level, thus providing no support for the increased intake or compensation hypotheses. We discuss these findings in light of the ecological causes and consequences of competition-induced plasticity in SMR.

Competition-driven changes in metabolic rates have been interpreted as responses to density-dependent shifts in foraging rates or locomotor activity^14^. This cannot be applied to our study, because the influence of locomotor activity on SMR variation was minor at both group and individual levels. In growing juveniles, SMR estimates are often confounded by the energy costs of digestion or somatic growth^9^. In our study, newts were starved for 6 days prior to respirometry trials, which period is longer than the duration in newts of the postprandial metabolic response (specific dynamic action)^23^. In addition, *I. alpestris* in heterospecific pairs and with larger conspecifics grew at similar rates while SMR increased in the former group only. The within-group trait correlation provides no support for the association between SMR and growth rates in *I*. *alpestris* from heterospecific pairs. Hence, the contribution of locomotor activity and growth rates to SMR plasticity appears minor in juvenile newts.

Alternatively, the competition-induced plasticity in SMR results from a link between SMR and hormonal profiles^24^. In salamanders, the presence of con- or heterospecific pheromones increases plasma corticosterone levels^25^, which in turn elevate SMR^26^. If this explanation holds, then our results suggest that competition-induced stress has (i) amplifying effects on SMR in the dominant species during heterospecific interactions, and (ii) species-specific influences on the relationship between SMR and growth rate. Both possibilities provide interesting research topics for future studies.

What are the ecological implications of a competition-induced shift in SMR? Because the ecological importance of SMR is context dependent^8^, it is difficult to judge the beneficial or detrimental consequences of this plastic response. If SMR is positively associated with aerobic capacity (the increased intake hypothesis), a high SMR may also be related to increased dominance and aggressiveness^10, 27^. That would provide advantages over metabolically slower individuals in competitive interactions. Indeed, greater aggressiveness towards heterospecifics rather than conspecifics has been reported in other juvenile salamanders^20, 21, 28^. Newts, however, lower their SMR after transition from the aquatic to terrestrial phase^29^ or from the active season to wintering^30^, suggesting that low maintenance costs are important for their economic lifestyle. From this viewpoint, elevated SMR constitutes a previously hidden cost of heterospecific competitive interactions rather than an advantage.

Competitive interactions with heterospecifics affected SMR in the larger species. The causes of this asymmetric plastic response are yet to be determined (see above). Although theory assumes an increase in metabolic rate due to the interference^31^, the metabolic rate of the competitively dominant species was unrelated to locomotor activity level. Clearly, increased SMR represents extra energy costs of heterospecific interactions, which were independent not only of locomotor activity but also of such other confounding factors as body size and growth rate. This suggests that the competitive advantage of larger size may be counterbalanced by higher maintenance costs in the dominant species. Accordingly, the asymmetric plastic response in SMR may complicate predictions of the outcome from heterospecific interactions in a stochastic environment.

The intensity and costs of heterospecific competitive interactions are thought to have been similar to those of conspecific interactions^16, 17^. The present study shows that the presence of heterospecifics, but not of conspecifics, induced plastic shifts in SMR. This finding has at least two important ecological implications. First, heterospecific competition may be mediated not only through variation in exploitation of resources and direct interference but also through elevated maintenance costs. These costs may be substantial. Our analysis showed that the presence of heterospecifics increases mean SMR by about 27% more than does the presence of conspecific individuals. Under conditions of limited resources availability (the compensation hypothesis), such costs may affect juvenile survival, and, accordingly, the population dynamics of the dominant species. Second, heterospecific competitive interactions may contribute to the unexplained variation in energy metabolism scaling in relation to body mass and temperature^32^. Hence, the previous experience of heterospecific interactions in measured individuals should be taken into account in collecting data for macroecological analyses. Further research on the mechanisms and adaptive significance of the competition-induced metabolic plasticity will provide more insight into the mutual effect of this fundamental biological rate and these ecological processes.

## Methods

### Animals

Juvenile newts were obtained from eggs of field-captured females (*n* = 25 per species) originating from two populations near Jihlava, Czech Republic. Larvae developed at natural densities (60 individuals m^−2^)^33^ under semi-natural conditions. After metamorphosis (16 July–4 August 2015), juveniles (*n* = 128; body mass [mean ± s.d.]: *I. alpestris*: 0.31 ± 0.09 g; *L. vulgaris*: 0.19 ± 0.06 g) were weighed (to precision 0.001 g; KERN EG, Balingen, Germany), marked with fluorescent elastomers (North-West Marine Technology, Shaw Island, USA), and haphazardly divided among three groups: singles, conspecific pairs, and heterospecific pairs (Fig. 1). Singles or pairs were placed in plastic tanks (16 × 9 × 14 cm; *n* = 80) equipped with water-saturated filter paper as a substrate and one dry beech leaf as shelter. Tanks were slightly inclined to provide some free well water on one side. Water availability was checked daily and refilled with deionized water as needed. Tanks were placed in a temperature- and photoperiod-controlled room with temperature in the range 12–22 °C and with a 12:12 h (light:dark) regime. Room air temperatures covered the temperature range that newts commonly experience in the field^34^. Newts were fed with an equal amount (0.02 g of wet mass per individual) of live *Tubifex* worms at three-day intervals, except for 6 days prior to metabolic trials. After 30–31 days, all individuals were reweighed. Growth rate (mg day^−1^) was calculated as the difference between final and original body mass divided by number of days of the experiment. Con- and heterospecific competition was measured as the difference in growth rate between paired and separately reared individuals of a given species. The influence of con- and heterospecific competitive interactions on (a) standard metabolic rates (minimum oxygen consumption), (b) somatic growth rates, and (c) spontaneous locomotor activity (distance moved during 30 min) were assessed in the juvenile newts *Ichthyosaura alpestris* and *Lissotriton vulgaris*. Metabolic and growth rates are body size-corrected means from a general linear model. Values are presented as means ± s.e.m. Groups are identified as follows: H, individuals from heterospecific pairs; C-B, larger conspecifics; C-S, smaller conspecifics; S, singles. With the exception of three pairs, *I. alpestris* individuals were larger than *L. vulgaris* individuals, and thus newts in heterospecific categories were not divided according to body size. All experiments were performed in accordance with relevant guidelines and regulations. This research was approved by the Institution of Vertebrate Biology’s Animal Ethics Committee (permit 14/2013). The Environment Department of the Regional Authority of Vysočina, Czech Republic, issued the permission to capture newts (KUJI 224/2013).

### Metabolic assays

Metabolic rate was measured using intermittent aerial respirometry. We used a nine-channel (eight chambers and baseline) respirometry system (Sable Systems, Las Vegas, NV, USA). Incurrent CO_2_- and H_2_O-free air (soda lime–silica gel and Drierite–Ascarite–Drierite gas scrubbers) was pushed by a mass-flow, meter-controlled air pump (120 ± 1 ml min^−1^). To minimize evaporative water loss of juvenile newts, air was rehumidified using Nafion^TM^ tubing (ME Series, Perma Pure, Toms River, NJ, USA) submerged in distilled water (18 ± 0.5 °C) before entering a respirometry chamber. We used a computer-controlled baselining unit and multiplexer (RM-8, Sable Systems) for automatic switching of air flow among channels. Custom-made respirometry chambers (30 ml) were submerged in a cooled water bath at 18 ± 0.3 °C. The chosen temperature was within the preferred temperature range of both species^35^. Excurrent air was passed through the water vapour analyser (RH-300, Sable Systems, Las Vegas, NV, USA), Nafion^TM^ dryer (MD Series, Perma Pure), CO_2_ analyser (FoxBox-C, Sable Systems), gas scrubber (soda lime–silica gel–Drierite), and O_2_ analyser (FoxBox-C), respectively. We used a high-resolution converter (UI-2, Sable Systems) to convert analogue analyser outputs into digital signals. To prevent water condensation inside the respirometry system, room temperature was maintained at 23 ± 2 °C. Verification of the respirometry system and calibration of analysers was in accordance with previously published procedures^36^.

Newts were starved for 6 days prior to measurements to attain their postabsorptive state^23^. After weighing (to precision 0.001 g; KERN EG, Balingen, Germany), newts were individually placed in a respirometry chamber. Because newts are predominantly crepuscular and nocturnal, metabolic trials were performed during daytime (8:00–19:00). Each trial lasted 5 h. Newt activity in the chamber was continuously monitored using a digital video camera (5 fps). The number of locomotor activity episodes was recorded using a motion video detector (5 s resolution). Respirometry chambers were flushed twice per hour (enclosure time = 1,679 s), which means we obtained ten measures of oxygen consumption per individual. We used the lowest oxygen consumption of a non-active individual (>95% of enclosure time) as the estimate of SMR.

Oxygen consumption was calculated from peak areas (integrals) for sample rates of O_2_ consumption (*M*_s_O_2_) divided by chamber enclosure time^37^. We used the following equation to calculate *M*_s_O_2_: *M*_s_O_2_ = *FR*(*F*_i_O_2_ − *F*_e_O_2_)/(1 − *F*_e_O_2_), where *FR* is the incurrent flow rate (ml h^−1^), *F*_e_O_2_ is the fractional concentration of excurrent O_2_, and *F*_i_O_2_ is the fractional concentration of incurrent O_2_. Data acquisition and calculations were performed using Expedata software (version 1.3.3, Sable Systems).

### Activity assays

We measured the spontaneous locomotor activity of newts as distance moved within a circular experimental arena (140 × 10 mm; *n* = 9) during 30 min. Each newt was placed in the arena individually 1 min before the activity trial. Arenas were covered with transparent acrylic to prevent escape and to minimize water loss of experimental subjects. Newt position was continuously recorded (3.75 fps) using an automated tracking system (Ethovision XT, Noldus, Wageningen, Netherlands). Trials were performed in darkness under infrared lighting at 18 ± 1 °C. Before each use, glass arenas were thoroughly washed in 95% ethanol to eliminate con- and heterospecific chemical cues.

### Statistical analyses

We used a general linear model (GLM) to test the effect of con- and heterospecific pairing on SMR and growth rates in each species (Table S2). Because SMR and growth rates are body size-dependent, we added final (SMR) and initial (growth rate) body mass as covariates to the model. Ranges of body mass values have little overlap between species, which violates the basic assumption for the use of covariates. In addition, because of the heterospecific pairing, the degrees of freedom would be artificially inflated within species groups. Hence, we applied the model separately for each species. Values are presented as body-mass adjusted (least squares) means ± s.e.m as obtained from the respective GLM. Because the sample sizes used precluded judging of the normality assumption, we used a permutation approach (9999 permutations) to obtain exact *P*-values of statistical tests using the PERMANOVA package in Primer (version 6.1.16, PRIMER-E Ltd, Lutton, UK). We reduced the false discovery rate in multiple comparison tests using the Benjamini-Hochberg procedure^38^. The ordinary GLM modelling and trait association tests (partial Spearman correlation) were performed using JMP (version 9.0.1, SAS Institute, Cary, USA) and the “ppcorr” package in R (R Foundation for Statistical Computing, Vienna, Austria), respectively. Datasets supporting this article are included in the electronic Supplementary Material, Table S4.

## Electronic supplementary material

Supplementary Information

## References

[1] Speakman, J. R. *Doubly labelled water: theory and practice* (London: Chapman and Hall, 1997).

[2] Nilsson JA (2002). Metabolic consequences of hard work. Proc. R. Soc. B-Biol. Sci..

[3] Chappell MA, Garland T, Robertson GF, Saltzman W (2007). Relationships among running performance, aerobic physiology and organ mass in male Mongolian gerbils. J. Exp. Biol..

[4] Biro PA, Stamps JA (2010). Do consistent individual differences in metabolic rate promote consistent individual differences in behavior?. Trends Ecol. Evol..

[5] Nagy KA, Girard IA, Brown TK (1999). Energetics of free-ranging mammals, reptiles, and birds. Ann. Rev. Nutr..

[6] Artacho P, Nespolo RF (2009). Natural selection reduces energy metabolism in the garden snail, *Helix aspersa* (*Cornu aspersum*). Evolution.

[7] Boratynski Z, Koteja P (2010). Sexual and natural selection on body mass and metabolic rates in free-living bank voles. Funct. Ecol..

[8] Burton T, Killen SS, Armstrong JD, Metcalfe NB (2011). What causes intraspecific variation in resting metabolic rate and what are its ecological consequences?. Proc. R. Soc. B-Biol. Sci.

[9] Speakman JR, Krol E, Johnson MS (2004). The functional significance of individual variation in basal metabolic rate. Physiol. Biochem. Zool..

[10] Careau V, Thomas D, Humphries MM, Reále D (2008). Energy metabolism and animal personality. Oikos.

[11] Metcalfe NB, Taylor AC, Thorpe JE (1995). Metabolic rate, social status and life-history strategies in Atlantic salmon. Anim. Behav..

[12] Millidine K, Metcalfe NB, Armstrong J (2009). Presence of a conspecific causes divergent changes in resting metabolism, depending on its relative size. Proc. R. Soc. B-Biol. Sci..

[13] Hou C, Kaspari M, Vander Zanden HB, Gillooly JF (2010). Energetic basis of colonial living in social insects. Proc. Natl. Acad. Sci. USA.

[14] DeLong JP, Hanley TC, Vasseur DA (2014). Competition and the density dependence of metabolic rates. J. Anim. Ecol..

[15] Sloman KA, Motherwell G, O’Connor KI, Taylor AC (2000). The effect of social stress on the standard metabolic rate (SMR) of brown trout. Salmo trutta. Fish Physiol. Biochem..

[16] Peiman KS, Robinson BW (2010). Ecology and evolution of resource-related heterospecific aggression. Q. Rev. Biol..

[17] Grether GF (2013). The evolutionary consequences of interspecific aggression. Ann. N.Y. Acad. Sci..

[18] Ros AFH, Vullioud P, Bruintjes R, Vallat A, Bshary R (2014). Intra- and interspecific challenges modulate cortisol but not androgen levels in a year-round territorial damselfish. J. Exp. Biol..

[19] Van Buskirk J (2007). Body size, competitive interactions, and the local distribution of *Triturus* newts. J. Anim. Ecol..

[20] Walls SC (1990). Interference competition in postmetamorphic salamanders: Interspecific differences in aggression by coexisting species. Ecology.

[21] Smyers SD, Rubbo MJ, Townsend VR, Swart CC (2002). Intra- and interspecific characterizations of burrow use and defense by juvenile ambystomatid salamanders. Herpetologica.

[22] Rosenfeld J, Van Leeuwen T, Richards J, Allen D (2015). Relationship between growth and standard metabolic rate: measurement artefacts and implications for habitat use and life-history adaptation in salmonids. J. Anim. Ecol..

[23] Gvoždík L, Kristín P (2017). Economic thermoregulatory response explains mismatch between thermal physiology and behavior in newts. J. Exp. Biol..

[24] Killen SS, Marras S, Metcalfe NB, McKenzie DJ, Domenici P (2013). Environmental stressors alter relationships between physiology and behaviour. Trends Ecol. Evol..

[25] Schubert SN (2009). Exposure to pheromones increases plasma corticosterone concentrations in a terrestrial salamander. Gen. Comp. Endocrinol..

[26] Wack CL (2012). Elevated plasma corticosterone increases metabolic rate in a terrestrial salamander. Comp. Biochem. Physiol. A-Mol. Integr. Physiol..

[27] Mathot KJ, Dingemanse NJ (2015). Energetics and behavior: unrequited needs and new directions. Trends Ecol. Evol..

[28] Nussbaum SE, Ousterhout BH, Semlitsch RD (2016). Agonistic behavior and resource defense among sympatric juvenile pond breeding salamanders. J. Herpetol..

[29] Kristín P, Gvoždík L (2014). Aquatic-to-terrestrial habitat shift reduces energy expenditure in newts. J. Exp. Zool. A-Ecol. Genet. Physiol..

[30] Podhajský L, Gvoždík L (2016). Variation in winter metabolic reduction between sympatric amphibians. Comp. Biochem. Physiol. A-Mol. Integr. Physiol..

[31] Zhang L, Andersen KH, Dieckmann U, Brännström A (2015). Four types of interference competition and their impacts on the ecology and evolution of size-structured populations and communities. J. Theor. Biol..

[32] Gillooly JF, Brown JH, West GB, Savage VM, Charnov EL (2001). Effects of size and temperature on metabolic rate. Science.

[33] Van Buskirk J, Schmidt BR (2000). Predator-induced phenotypic plasticity in larval newts: trade-offs, selection, and variation in nature. Ecology.

[34] Šamajová P, Gvoždík L (2010). Inaccurate or disparate temperature cues? Seasonal acclimation of terrestrial and aquatic locomotor capacity in newts. Funct. Ecol..

[35] Balogová M, Gvoždík L (2015). Can newts cope with the heat? Disparate thermoregulatory strategies of two sympatric species in water. PLoS ONE.

[36] Kristín P, Gvoždík L (2012). Influence of respirometry methods on intraspecific variation in standard metabolic rates in newts. Comp. Biochem. Physiol. A-Mol. Integr. Physiol.

[37] Lighton, J. R. B. *Measuring metabolic rates: a manual for scientists* (Oxford: Oxford University Press, 2008).

[38] Benjamini Y, Hochberg Y (1995). Controlling the false discovery rate: a practical and powerful approach to multiple testing. J. R. Stat. Soc. B..

