# Supplementary material for: Costly neighbours: Heterospecific competitive interactions increase metabolic rates in dominant species
Source: Sci Rep. 2017 Jul 12;7:5177. doi: 10.1038/s41598-017-05485-9 (PMC5507852; doi:10.1038/s41598-017-05485-9)
Supplement: Supplementary file 1 — Supplementary Information [file 41598_2017_5485_MOESM1_ESM.doc]

Electronic supplementary material from “Costly neighbors: Heterospecific competitive interactions increase metabolic rates in dominant species”

Matouš Janča^1^, Lumír Gvoždík^2*^

^1^Department of Botany and Zoology, Masaryk University, Kotlářská 267/2, 611 37 Brno, Czech Republic

^2^Institute of Vertebrate Biology AS CR, Květná 8, 60365 Brno, Czech Republic

^*^Corresponding author:

Lumír Gvoždík

Institute of Vertebrate Biology AS CR

Research Facility “Studenec”

Studenec 122

67502 Koněšín

Czech Republic

Tel.: +420568422763; Fax: +420568423121

[gvozdik@brno.cas.cz](mailto:gvozdik@brno.cas.cz)

Table S1. General linear model parameters with statistical tests of their differences from zero. Models tested the influence of con- and heterospecific competitive interactions on minimum oxygen consumption, somatic growth, and voluntary activity in juvenile newts, *Ichthyosaura alpestris* and *Lissotriton vulgaris*. Note that factor significance was inferred from permutation test results (see main text details).

| Factor | | *I. alpestris* | | | |  | *L. vulgaris* | | | |
| --- | --- | --- | --- | --- | --- | --- | --- | --- | --- | --- |
|  |  | Parameter | SE | *t* | *P* |  | Parameter | SE | *t* | *P* |
| Oxygen consumption  (ml h^-1^) | |  |  |  |  |  |  |  |  |  |
|  | Intercept | 0.0024316 | 0.009039 | 0.27 | 0.7891 |  | -0.011244 | 0.006928 | -1.62 | 0.1113 |
|  | TRT[C_B] | -0.005159 | 0.003072 | -1.68 | 0.0994 |  | 0.0012956 | 0.002248 | 0.58 | 0.5672 |
|  | TRT[C_S] | 0.0004918 | 0.003268 | 0.15 | 0.8810 |  | -0.003256 | 0.002314 | -1.41 | 0.1659 |
|  | TRT[H] | 0.0094323 | 0.002944 | 3.20 | **0.0024** |  | 0.0039167 | 0.002081 | 1.88 | 0.0660 |
|  | BM_end | 0.094357 | 0.023869 | 3.95 | **0.0002** |  | 0.1725473 | 0.035918 | 4.80 | **<.0001** |
| Growth rate (mg d^-1^) | |  |  |  |  |  |  |  |  |  |
|  | Intercept | 0.0031178 | 0.000372 | 8.39 | **<.0001** |  | 0.001346 | 0.000407 | 3.30 | **0.0017** |
|  | TRT[C_B] | 0.0003885 | 0.00018 | 2.16 | **0.0351** |  | -1.032e-5 | 0.000181 | -0.06 | 0.9548 |
|  | TRT[C_S] | -0.000501 | 0.000186 | -2.69 | **0.0095** |  | 1.74e-6 | 0.000191 | 0.01 | 0.9928 |
|  | TRT[H] | 0.0004407 | 0.000179 | 2.46 | **0.0170** |  | 0.0000347 | 0.000177 | 0.20 | 0.8455 |
|  | BM_start | -0.006504 | 0.00116 | -5.60 | **<.0001** |  | -0.006284 | 0.002099 | -2.99 | **0.0042** |
| Locomotor activity  (cm 30 min^-1^) | |  |  |  |  |  |  |  |  |  |
|  | Intercept | 1042.0133 | 107.3032 | 9.71 | **<.0001** |  | 1375.9455 | 93.02358 | 14.79 | **<.0001** |
|  | TRT[C_B] | -33.68715 | 183.7059 | -0.18 | 0.8552 |  | 450.55302 | 168.2228 | 2.68 | **0.0099** |
|  | TRT[C_S] | 205.31601 | 187.9788 | 1.09 | 0.2796 |  | 54.243024 | 168.2228 | 0.32 | 0.7484 |
|  | TRT[H] | -0.940873 | 187.9788 | -0.01 | 0.9960 |  | -22.87276 | 159.6634 | -0.14 | 0.8866 |

TRT: treatment.

C_B: bigger individuals in conspecific pairs.

C_S: smaller individuals in conspecific pairs.

H: heterospecific pairs.

BM_end: body mass at competition experiment end.

BM_start: body mass at competition experiment beginning.

Table S2. Spearman partial correlation coefficients (*r*_s_) with *P*-values for the association between standard metabolic rate (SMR) and growth rate, and between SMR and spontaneous (locomotor) activity in juvenile newts *Ichthyosaura alpestris* and *Lissotriton vulgaris*, exposed to con- and heterospecific competitive interactions. All bivariate associations were tested with the removed effect of other variables (initial body mass, final body mass, growth rate, or activity).

| Experimental group | *I. alpestris* | | |  | *L. vulgaris* | | |
| --- | --- | --- | --- | --- | --- | --- | --- |
|  | *n* | *r_s_* | *P* |  | *n* | *r_s_* | *P* |
| SMR vs. Growth rate |  |  |  |  |  |  |  |
| Heterospecific | 14 | -0.46 | 0.15 |  | 13 | -0.03 | 0.93 |
| Conspecific - big | 13 | 0.19 | 0.61 |  | 11 | 0.11 | 0.80 |
| Conspecific - small | 13 | -0.25 | 0.49 |  | 11 | -0.48 | 0.23 |
| Single | 14 | -0.10 | 0.76 |  | 17 | -0.10 | 0.74 |
| SMR vs. Locomotor activity |  |  |  |  |  |  |  |
| Heterospecific | 14 | 0.50 | 0.12 |  | 13 | 0.24 | 0.50 |
| Conspecific - big | 13 | -0.10 | 0.78 |  | 11 | -0.17 | 0.68 |
| Conspecific - small | 13 | 0.24 | 0.50 |  | 11 | -0.11 | 0.79 |
| Single | 14 | -0.14 | 0.69 |  | 17 | 0.46 | 0.10 |

Table S3. Descriptive statistics of body mass, oxygen consumption, locomotor activity, and growth rate in juvenile newts exposed to con- and heterospecific competitive interactions.

| Group | Body mass (g) | | | Oxygen consumption  (ml h^-1^) | | | Locomotor activity  (cm 30 min^-1^) | | | Growth rate  (mg d^-1^) | | |
| --- | --- | --- | --- | --- | --- | --- | --- | --- | --- | --- | --- | --- |
|  | *n* | Mean | SE | *n* | Mean | SE | *n* | Mean | SE | *n* | Mean | SE |
| *I. alpestris* |  |  |  |  |  |  |  |  |  |  |  |  |
| C_B | 15 | 0.420 | 0.020 | 13 | 0.036 | 0.002 | 15 | 1008.3 | 122.6 | 15 | 1.268 | 0.130 |
| C_S | 15 | 0.310 | 0.010 | 13 | 0.032 | 0.002 | 14 | 1247.3 | 282.8 | 15 | 0.974 | 0.280 |
| H | 14 | 0.400 | 0.026 | 14 | 0.050 | 0.006 | 14 | 1041.1 | 227.1 | 14 | 1.559 | 0.350 |
| S | 15 | 0.367 | 0.015 | 14 | 0.032 | 0.002 | 15 | 871.3 | 206.1 | 15 | 0.654 | 0.210 |
| *L. vulgaris* |  |  |  |  |  |  |  |  |  |  |  |  |
| C_B | 12 | 0.206 | 0.009 | 11 | 0.025 | 0.002 | 12 | 1826.5 | 270.1 | 12 | 0.066 | 0.160 |
| C_S | 12 | 0.178 | 0.011 | 11 | 0.015 | 0.003 | 12 | 1430.2 | 157.0 | 12 | 0.366 | 0.250 |
| H | 14 | 0.215 | 0.021 | 13 | 0.026 | 0.003 | 14 | 1353.1 | 163.5 | 14 | 0.044 | 0.300 |
| S | 18 | 0.193 | 0.010 | 17 | 0.020 | 0.003 | 18 | 894.0 | 148.7 | 18 | 0.175 | 0.110 |

C_B: bigger individuals in conspecific pairs.

C_S: smaller individuals in conspecific pairs.

H: heterospecific pairs.

S: singles

Table S4. Raw data of minimum oxygen consumption, somatic growth rates, and spontaneous activity (distance moved) in juvenile newts, *Ichthyosaura alpestris* and *Lissotriton vulgaris*, exposed to con- and heterospecific competitive interactions.

| Species | Treatment | Tank | BM_start | BM_end | VO2 | VO2_  definition | Distance_  moved | Growth_  rate |
| --- | --- | --- | --- | --- | --- | --- | --- | --- |
| vulgaris | single | 1 | 0.160 | 0.154 | 0.00767 | SMR | 31.47 | -0.09677 |
| vulgaris | single | 2 | 0.150 | 0.192 | 0.01580 | SMR | 54.16 | 0.67742 |
| vulgaris | conspecific_bigger | 3 | 0.180 | 0.214 | 0.02775 | SMR | 174.41 | 0.54839 |
| vulgaris | conspecific_smaller | 3 | 0.110 | 0.164 | 0.01595 | SMR | 186.17 | 0.87097 |
| vulgaris | single | 4 | 0.140 | 0.151 | 0.00923 | SMR | 15.89 | 0.17742 |
| vulgaris | single | 5 | 0.160 | 0.159 | 0.01042 | SMR | 89.89 | -0.01613 |
| vulgaris | single | 6 | 0.200 | 0.189 | 0.01790 | SMR | 135.47 | -0.17742 |
| vulgaris | conspecific_smaller | 7 | 0.122 | 0.130 | 0.01374 | SMR | 92.58 | 0.12903 |
| vulgaris | conspecific_bigger | 7 | 0.146 | 0.167 | 0.01655 | SMR | 62.62 | 0.33871 |
| vulgaris | single | 9 | 0.163 | 0.137 | 0.01248 | SMR | 127.51 | -0.41936 |
| vulgaris | single | 10 | 0.087 | 0.113 | 0.01390 | SMR | 235.00 | 0.41936 |
| vulgaris | single | 11 | 0.129 | 0.167 | 0.01815 | SMR | 182.31 | 0.61290 |
| vulgaris | single | 13 | 0.169 | 0.171 | 0.01177 | SMR | 137.26 | 0.03333 |
| alpestris | heterospecific | 14 | 0.344 | 0.412 | 0.04685 | SMR | 193.03 | 1.13333 |
| vulgaris | heterospecific | 14 | 0.221 | 0.194 | 0.02057 | SMR | 177.76 | -0.45000 |
| vulgaris | single | 15 | 0.210 | 0.174 | 0.01929 | SMR | 57.78 | -0.60000 |
| alpestris | heterospecific | 16 | 0.342 | 0.400 | 0.07490 | SMR | 80.07 | 0.96667 |
| vulgaris | heterospecific | 16 | 0.148 | 0.157 | 0.01762 | SMR | 189.38 | 0.15000 |
| vulgaris | conspecific_bigger | 17 | 0.213 | 0.248 | 0.02770 | SMR | 196.96 | 0.59322 |
| vulgaris | conspecific_smaller | 17 | 0.167 | 0.199 | 0.00353 | SMR | 130.70 | 0.54237 |
| vulgaris | conspecific_smaller | 18 | 0.171 | 0.200 | 0.00348 | SMR | 130.62 | 0.49153 |
| vulgaris | conspecific_bigger | 18 | 0.190 | 0.202 | 0.04334 | SMR | 153.97 | 0.20339 |
| alpestris | heterospecific | 19 | 0.307 | 0.395 | 0.02037 | SMR | 66.50 | 1.49153 |
| vulgaris | heterospecific | 19 | 0.200 | 0.180 | 0.02403 | SMR | 129.74 | -0.33898 |
| alpestris | heterospecific | 20 | 0.386 | 0.540 | 0.09185 | SMR | 246.49 | 2.61017 |
| vulgaris | heterospecific | 20 | 0.182 | 0.148 | 0.01260 | SMR | 79.67 | -0.57627 |
| alpestris | heterospecific | 21 | 0.261 | 0.350 | 0.01581 | SMR | 19.25 | 1.50848 |
| vulgaris | heterospecific | 21 | 0.202 | 0.208 | 0.02153 | SMR | 222.38 | 0.10170 |
| alpestris | heterospecific | 22 | 0.311 | 0.412 | 0.02672 | SMR | 67.17 | 1.71186 |
| vulgaris | heterospecific | 22 | 0.227 | 0.216 | 0.04420 | SMR | 241.73 | -0.18644 |
| vulgaris | single | 23 | 0.167 | 0.230 | 0.02134 | SMR | 88.66 | 1.06780 |
| vulgaris | conspecific_smaller | 24 | 0.172 | 0.154 | 0.00151 | SMR | 132.61 | -0.30509 |
| vulgaris | conspecific_bigger | 24 | 0.223 | 0.238 | 0.03044 | SMR | 288.89 | 0.25424 |
| alpestris | heterospecific | 25 | 0.154 | 0.231 | 0.04267 | SMR | 229.33 | 1.30509 |
| vulgaris | heterospecific | 25 | 0.307 | 0.476 | 0.06003 | RMR | 101.61 | 2.86441 |
| alpestris | heterospecific | 26 | 0.228 | 0.171 | 0.02782 | SMR | 5.09 | -0.96610 |
| vulgaris | heterospecific | 26 | 0.299 | 0.204 | 0.01786 | SMR | 45.00 | -1.61017 |
| alpestris | heterospecific | 27 | 0.180 | 0.456 | 0.05162 | SMR | 79.67 | 4.67797 |
| vulgaris | heterospecific | 27 | 0.110 | 0.223 | 0.02754 | SMR | 87.02 | 1.91525 |
| alpestris | heterospecific | 28 | 0.359 | 0.454 | 0.03800 | SMR | 16.15 | 1.61017 |
| vulgaris | heterospecific | 28 | 0.208 | 0.241 | 0.03452 | SMR | 182.99 | 0.55932 |
| alpestris | heterospecific | 29 | 0.222 | 0.395 | 0.03437 | SMR | 212.35 | 2.93220 |
| vulgaris | heterospecific | 29 | 0.235 | 0.200 | 0.03438 | SMR | 168.82 | -0.59322 |
| alpestris | heterospecific | 30 | 0.384 | 0.444 | 0.06931 | SMR | 152.33 | 1.01695 |
| vulgaris | heterospecific | 30 | 0.249 | 0.211 | 0.03539 | SMR | 100.06 | -0.64407 |
| vulgaris | conspecific_smaller | 31 | 0.122 | 0.275 | 0.05213 | RMR | 173.89 | 2.59322 |
| vulgaris | conspecific_bigger | 31 | 0.146 | 0.180 | 0.01792 | SMR | 193.91 | 0.57627 |
| vulgaris | single | 34 | 0.196 | 0.200 | 0.01324 | SMR | 86.35 | 0.06557 |
| vulgaris | single | 35 | 0.211 | 0.235 | 0.02947 | SMR | 42.17 | 0.39344 |
| vulgaris | single | 36 | 0.169 | 0.217 | 0.04887 | RMR | 153.65 | 0.78689 |
| vulgaris | conspecific_bigger | 37 | 0.204 | 0.197 | 0.01756 | SMR | 149.42 | -0.11475 |
| vulgaris | conspecific_smaller | 37 | 0.162 | 0.155 | 0.02174 | SMR | 264.10 | -0.11475 |
| alpestris | conspecific_bigger | 39 | 0.428 | 0.497 | 0.08557 | RMR | 36.88 | 1.15000 |
| alpestris | conspecific_smaller | 39 | 0.191 | 0.275 | 0.03529 | SMR | 68.27 | 1.40000 |
| vulgaris | single | 40 | 0.275 | 0.261 | 0.04264 | SMR | 107.12 | -0.23333 |
| vulgaris | conspecific_bigger | 42 | 0.161 | 0.159 | 0.03091 | SMR | 144.90 | -0.03333 |
| vulgaris | conspecific_smaller | 42 | 0.143 | 0.164 | 0.01382 | SMR | 188.39 | 0.35000 |
| alpestris | conspecific_bigger | 43 | 0.372 | 0.437 | 0.03800 | SMR | 70.16 | 1.08333 |
| alpestris | conspecific_smaller | 43 | 0.321 | 0.398 | 0.03203 | SMR | 179.92 | 1.28333 |
| vulgaris | conspecific_smaller | 44 | 0.157 | 0.156 | 0.02377 | SMR | 121.03 | -0.01667 |
| vulgaris | conspecific_bigger | 44 | 0.250 | 0.233 | 0.05917 | RMR | 19.62 | -0.28333 |
| alpestris | conspecific_bigger | 45 | 0.253 | 0.385 | 0.03397 | SMR | 210.20 | 2.20000 |
| alpestris | conspecific_smaller | 45 | 0.499 | 0.351 | 0.04182 | SMR | 40.63 | -2.46667 |
| vulgaris | single | 46 | 0.221 | 0.246 | 0.01686 | SMR | 6.30 | 0.40984 |
| vulgaris | single | 47 | 0.303 | 0.270 | 0.05815 | SMR | 37.15 | -0.54098 |
| alpestris | single | 48 | 0.547 | 0.476 | 0.03104 | SMR | 60.90 | -1.20339 |
| alpestris | heterospecific | 49 | 0.527 | 0.528 | 0.08072 | SMR | 33.03 | 0.01695 |
| vulgaris | heterospecific | 49 | 0.229 | 0.199 | 0.03469 | SMR | 98.11 | -0.50848 |
| alpestris | single | 50 | 0.482 | 0.486 | 0.04200 | SMR | 9.87 | 0.06780 |
| vulgaris | single | 51 | 0.169 | 0.204 | 0.01857 | SMR | 21.12 | 0.59322 |
| alpestris | single | 52 | 0.420 | 0.433 | 0.03357 | SMR | 17.82 | 0.22034 |
| vulgaris | conspecific_bigger | 53 | 0.193 | 0.210 | 0.02543 | SMR | 333.88 | 0.28333 |
| vulgaris | conspecific_smaller | 53 | 0.148 | 0.199 | 0.02548 | SMR | 146.94 | 0.85000 |
| alpestris | single | 54 | 0.272 | 0.343 | 0.02650 | SMR | 15.99 | 1.18333 |
| alpestris | single | 55 | 0.222 | 0.290 | 0.02573 | SMR | 75.08 | 1.13333 |
| alpestris | single | 56 | 0.404 | 0.382 | 0.03672 | SMR | 30.75 | -0.36667 |
| alpestris | single | 57 | 0.397 | 0.394 | 0.03293 | SMR | 146.05 | -0.05000 |
| alpestris | conspecific_bigger | 58 | 0.439 | 0.477 | 0.08512 | RMR | 105.61 | 0.63333 |
| alpestris | conspecific_smaller | 58 | 0.327 | 0.356 | 0.05864 | RMR | 106.87 | 0.48333 |
| vulgaris | conspecific_smaller | 59 | 0.200 | 0.155 | 0.01549 | SMR | 83.70 | -0.76271 |
| vulgaris | conspecific_bigger | 59 | 0.181 | 0.175 | 0.01651 | SMR | 160.48 | -0.10170 |
| alpestris | single | 60 | 0.247 | 0.322 | 0.04219 | SMR | 121.40 | 1.27119 |
| alpestris | conspecific_bigger | 61 | 0.471 | 0.604 | 0.05043 | SMR | 117.90 | 2.25424 |
| alpestris | conspecific_smaller | 61 | 0.333 | 0.321 | 0.04832 | SMR | 182.93 | -0.20339 |
| alpestris | conspecific_smaller | 62 | 0.209 | 0.320 | 0.03139 | SMR | 209.89 | 1.88136 |
| alpestris | conspecific_bigger | 62 | 0.326 | 0.403 | 0.05444 | SMR | 159.15 | 1.30509 |
| alpestris | conspecific_smaller | 63 | 0.203 | 0.295 | 0.03060 | SMR | 47.84 | 1.53333 |
| alpestris | conspecific_bigger | 63 | 0.294 | 0.392 | 0.03056 | SMR | 133.82 | 1.63333 |
| alpestris | heterospecific | 64 | 0.298 | 0.407 | 0.07301 | SMR | 57.06 | 1.81667 |
| vulgaris | heterospecific | 64 | 0.161 | 0.157 | 0.01775 | SMR | 70.01 | -0.06667 |
| alpestris | single | 65 | 0.262 | 0.341 | 0.03030 | SMR | 210.58 | 1.31667 |
| alpestris | conspecific_smaller | 66 | 0.235 | 0.305 | 0.02909 | SMR | 117.73 | 1.16667 |
| alpestris | conspecific_bigger | 66 | 0.234 | 0.325 | 0.03852 | SMR | 41.38 | 1.51667 |
| vulgaris | conspecific_smaller | 67 | 0.201 | 0.187 | 0.02344 | SMR | 65.51 | -0.23729 |
| vulgaris | conspecific_bigger | 67 | 0.338 | 0.251 | 0.02312 | SMR | 312.74 | -1.47458 |
| alpestris | single | 68 | 0.280 | 0.346 | 0.02863 | SMR | 48.22 | 1.11864 |
| alpestris | conspecific_smaller | 69 | 0.254 | 0.326 | 0.03064 | SMR | 72.39 | 1.22034 |
| alpestris | conspecific_bigger | 69 | 0.262 | 0.342 | 0.02798 | SMR | 55.42 | 1.35593 |
| alpestris | single | 70 | 0.299 | 0.355 | 0.03640 | SMR | 171.78 | 0.94915 |
| alpestris | conspecific_smaller | 71 | 0.210 | 0.293 | 0.03053 | SMR | 154.21 | 1.40678 |
| alpestris | conspecific_bigger | 71 | 0.352 | 0.420 | 0.03559 | SMR | 96.51 | 1.15254 |
| alpestris | single | 72 | 0.350 | 0.350 | 0.02625 | SMR | 271.80 | 0.00000 |
| alpestris | single | 73 | 0.248 | 0.333 | 0.02716 | SMR | 45.55 | 1.44068 |
| alpestris | single | 74 | 0.290 | 0.366 | 0.06881 | RMR | 57.68 | 1.28814 |
| alpestris | single | 75 | 0.206 | 0.291 | 0.03312 | SMR | 23.52 | 1.44068 |
| alpestris | conspecific_bigger | 76 | 0.359 | 0.384 | 0.03084 | SMR | 66.04 | 0.42373 |
| alpestris | conspecific_smaller | 76 | 0.152 | 0.236 | 0.05126 | RMR | 428.83 | 1.42373 |
| alpestris | conspecific_bigger | 77 | 0.459 | 0.541 | 0.03706 | SMR | 77.89 | 1.38983 |
| alpestris | conspecific_smaller | 77 | 0.210 | 0.278 | 0.02566 | SMR |  | 1.15254 |
| alpestris | conspecific_bigger | 78 | 0.317 | 0.370 | 0.02841 | SMR | 84.18 | 0.89831 |
| alpestris | conspecific_smaller | 78 | 0.251 | 0.302 | 0.04485 | SMR | 61.79 | 0.86441 |
| alpestris | conspecific_smaller | 80 | 0.208 | 0.323 | 0.02185 | SMR | 30.36 | 1.94915 |
| alpestris | conspecific_bigger | 80 | 0.287 | 0.355 | 0.02878 | SMR | 146.01 | 1.15254 |
| alpestris | conspecific_smaller | 81 | 0.186 | 0.275 | 0.01921 | SMR | 44.62 | 1.50848 |
| alpestris | conspecific_bigger | 81 | 0.310 | 0.361 | 0.03184 | SMR | 111.33 | 0.86441 |

Treatment: single, newts reared separately; conspecific_bigger, bigger individuals in conspecific pairs; conspecific_smaller, smaller individuals in conspecific pairs; heterospecific, heterospecific pairs.

Tank: tank identity

Body_mass_start: body mass (g) at the beginning of the competition experiment. This body mass was used as the covariate in analysis of growth rates.

VO2: Minimum oxygen consumption (ml h^-1^) measured after the competition experiment.

VO2_definition: SMR, standard metabolic rate; RMR, routine metabolic rate (individuals were active during VO2 measurements). Note that only SMR values were used for statistical analyses.

Body_mass_end: body mass (g) at the end of the competition experiment. This body mass was used as the covariate in the analysis of SMR.

Distance_moved: Distance (cm) moved in experimental arena during 30 min trial of spontaneous activity.

Growth_rate: Somatic growth rates (mg d^-1^) calculated as the difference between initial and final body mass divided by the duration of the experiment (30 or 31 days).
